# The *AXEAP2* program for *K*β X-ray emission spectra analysis using artificial intelligence

**DOI:** 10.1107/S1600577523005684

**Published:** 2023-08-01

**Authors:** In-Hui Hwang, Shelly D. Kelly, Maria K. Y. Chan, Eli Stavitski, Steve M. Heald, Sang-Wook Han, Nicholas Schwarz, Cheng-Jun Sun

**Affiliations:** a X-ray Science Division, Argonne National Laboratory, Lemont, IL 60439, USA; b Center for Nanoscale Materials, Argonne National Laboratory, Lemont, IL 60439, USA; c National Synchrotron Light Source II, Brookhaven National Laboratory, NY 11973, USA; d Department of Physics Education and Institute of Fusion Science, Jeonbuk National University, Jeonju 54896, Republic of Korea; ESRF – The European Synchrotron, France

**Keywords:** *AXEAP*, XES, electron interaction, genetic algorithm, spin state

## Abstract

The processing and analysis of synchrotron data can be a complex task, requiring specialized expertise and knowledge. Our previous work addressed the challenge of X-ray emission spectrum (XES) data processing by developing a standalone application using unsupervised machine learning. However, the task of analyzing the processed spectra remains another challenge. Although the non-resonant *K*β XES of 3*d* transition metals are known to provide electronic structure information such as oxidation and spin state, finding appropriate parameters to match experimental data is a time-consuming and labor-intensive process. Here, a new XES data analysis method based on the genetic algorithm is demonstrated, applying it to Mn, Co and Ni oxides. This approach is also implemented as a standalone application, *Argonne X-ray Emission Analysis 2* (*AXEAP2*), which finds a set of parameters that result in a high-quality fit of the experimental spectrum with minimal intervention. *AXEAP2* is able to find a set of parameters that reproduce the experimental spectrum, and provide insights into the 3*d* electron spin state, 3*d*–3*p* electron exchange force and *K*β emission core-hole lifetime.

## Abstract 2

A methodology for analyzing 3*d* transition metal XES and a user-friendly program named *AXEAP2* are presented.

## 1. Introduction

The non-resonant *K*β X-ray emission spectrum (XES) of 3*d* transition metals is a widely used tool in the investigation of the electronic structure of materials of interest in condensed matter physics, coordination chemistry and catalysis (Vankó *et al.*, 2006 ▸; Lafuerza *et al.*, 2020 ▸; Kucheryavy *et al.*, 2016 ▸). The XES is a result of an electric-dipole-allowed 3*p* → 1*s* transition following an incident photon with sufficient energy to excite a 1*s* electron through the absorption process. The XES shape is known to highly depend on spin state and oxidation state. These characteristics are often collected as a complementary method to X-ray absorption spectroscopy (XAS) and are widely used to elucidate the unique structural and electronic information by comparing spectral shape with known standards samples (Bauer, 2014 ▸; Burkhardt *et al.*, 2017 ▸; Agote-Arán *et al.*, 2019 ▸; Lassalle-Kaiser *et al.*, 2017 ▸; Glatzel & Bergmann, 2005 ▸; Gretarsson *et al.*, 2013 ▸; Pelliciari *et al.*, 2017 ▸; Chin *et al.*, 2017 ▸).

Atomic multiplet theory and crystal field effects provide a solid theoretical foundation for explaining the XES spectra (Cowan, 1981 ▸; de Groot & Kotani, 2008 ▸). Atomic multiplet theory calculations are based on the interaction of two electrons and spin–orbital coupling in a many-body system. In 3*d* transition metals, the Coulomb and exchange force of 3*d*–3*d* and 3*d*–3*p* electrons are considered, which cause the *K*β emission line to split into a *K*β_1,3_ and a *K*β′ line. In the crystal field effect, the ligands and positively charged metal cation break the degeneracies of the 3*d* electron orbital and separate them into energy levels such as *t*_2*g*_ and *e*_*g*_, leading to differences in the number of pairing electrons and spin state. Usually, the high-spin (HS) spectrum has relatively prominent *K*β′ features compared with the low-spin (LS) spectrum, even if the number of 3*d* electrons is the same. Therefore, electron–electron interactions, the number of electrons and the spin state in the 3*d* orbital all play crucial roles in determining the spectral shape.

Our group recently developed a program, *Argonne X-ray Emission Analysis Package* (*AXEAP*) (Hwang *et al.*, 2022 ▸), that was able to dramatically increase the processing speed of raw data by using unsupervised machine learning. However, data analysis remained challenging due to the tedious and difficult process of finding parameters in multiplet spectral simulations that match experimental data, leading to a trial-and-error method for determining them. This problem occurs not only in XES but also in various fields trying to match theory and experimental data. To address this challenge artificial intelligence (AI), such as machine learning (ML) including deep learning, has been applied to data analysis (Zheng *et al.*, 2018 ▸; Timoshenko & Frenkel, 2019 ▸; Miyazato *et al.*, 2019 ▸; Benmore *et al.*, 2022 ▸). ML consists of numerical models that can predict output classes or values from input data. This method requires a lot of training data because model parameters must be trained to enable accurate prediction. However, there is insufficient experimental XES data with known ground truth to reliably train these ML models.

In this study, a methodology for analyzing 3*d* transition metal XES and a user-friendly program named *AXEAP2* are presented. The methodology includes three aspects. The first is the calculation of the XES, which is based on the *CTM4XAS* program (Stavitski & de Groot, 2010 ▸). The second is convolution after the XES calculation. The third part is parameter optimization driven by a genetic algorithm (GA), which is based upon an evolutionary metaphor that reflects the process of natural selection where the fittest individuals or parameters are selected repeatedly (Mitchell, 1996 ▸). This algorithm efficiently finds optimized values in multiple parameter spaces and is widely used to generate high-quality solutions for optimization problems (Terry *et al.*, 2021 ▸; Li *et al.*, 2017 ▸; Pettersson & Takahashi, 2021 ▸). In addition, we developed a viewer function to aid in the interpretation of results. The performance of the methodology was evaluated by analyzing the emission spectra of Mn, Co and Ni oxides.

## 2. XES measurement and experimental broadening calculation

To test the performance of *AXEAP2*, the XES of MnO, MnCO_3_, Mn_2_O_3_, CoO, LiCoO_2_ and LiNiO_2_ were measured at the 20-ID-C beamline of the Advanced Photon Source at Argonne National Laboratory, USA. The chemical characteristics of each sample are listed in Table 1 ▸ with reference to the literature (Fubini & Stone, 1983 ▸; Qiao *et al.*, 2013 ▸; Gamblin & Urch, 2001 ▸; Sicolo *et al.*, 2020 ▸; Montoro *et al.*, 1999 ▸). These samples were obtained from Aldrich without further purification and selected to compare the differences in the spectrum according to the local environment around the transition metal ion as follows.

(1) The same element but different number of electrons (MnO and Mn_2_O_3_).

(2) The same element and the same number of electrons (MnO and MnCO_3_).

(3) The same element but different number of electrons and different spin state (CoO and LiCoO_2_).

(4) A sample whose valency is not yet clear (LiNiO_2_).

(5) The same number of electrons but different spin state when the LiNiO_2_ is trivalent (CoO and LiNiO_2_).

A miniature X-ray emission spectrometer (miniXES) was employed to obtain high-resolution spectra within ∼1 eV (Solovyev *et al.*, 2021 ▸; Mattern *et al.*, 2012 ▸). The miniXES requires a micro-focused incident X-ray beam with a short working distance, flat analyzer crystals arranged on von Hamos geometry, and two-dimensional position-sensitive detector (2D-PSD) to collect energy-resolved fluorescence signal from the sample. The energies of monochromatic X-rays diffracted from a Si(111) double-crystal monochromator were calibrated to the first derivative peaks of the *K*-edges of Mn, Co and Ni foils. To focus the beam, Kirk­patrick–Baez mirrors were used, and the focused beam diameter at the sample position was about 50 µm. In order to diffract fluorescence emitted from the sample, eight analyzers were arranged side by side on a von-Hamos circle. Here, Ge(044), Ge(444) and Si(444) satisfying Bragg angles for the *K*β emission range of Mn, Co and Ni, respectively, were used as the analyzers. For the 2D-PSD, a DECTRIS 500K was used and placed at a specific distance according to the optical design. More details about the experimental setup, data acquisition and data processing method are given elsewhere (Hwang *et al.*, 2022 ▸).

**Figure 1:**
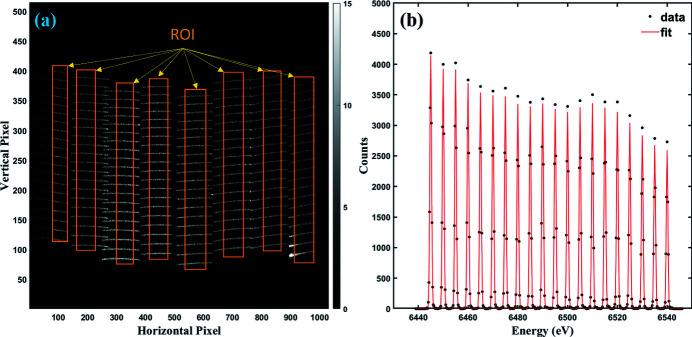
(*a*) An image of elastically scattered X-rays from MnO on the 2D-PSD for an incident energy range 6445–6540 eV at 5 eV intervals. The horizontal lines with high count rate (white) correspond to each energy and a ROI is set for each of the 20 lines arranged vertically. (*b*) An example of estimating the energy resolution elastic scattering line used for (*a*) calibration image with accompanying Gaussian fit.

The broadening of the spectrum, half width at half-maximum (HWHM), due to the 2D-PSD pixel resolution was determined by fitting elastic scattering lines with a Gaussian function. The resolution depends on the pixel size, optical geometry and analyzer (Solovyev *et al.*, 2021 ▸). The elastic scatterings were collected during the calibration. *AXEAP* provides the HWHM estimation and Fig. 1 ▸ shows an example of the calibration image and its line processed by *AXEAP*. The image shown in Fig. 1 ▸(*a*) is a merged image from 20 series scans corresponding to a Mn *K*β emission range of 6445 to 6540 eV for detector energy calibration. Each horizontal line in the eight regions of interests (ROIs) represents the location of the photons collected using monochromatic X-rays and eight Ge(044) analyzers for 60 s. The image shows that some horizontal lines overlap horizontally with adjacent horizontal lines, which are treated as unusable data occurring in the experimental setup. Using unsupervised ML, *AXEAP* efficiently identifies the overlapping region and recognizes it as unusable data. This allows it to exclude these areas from the ROI. Additionally, *AXEAP* is intelligently designed to exclude a small area near the boundary of the ROI, thereby preventing any potential noise data from being included in the analysis. When energy values are assigned to all pixels in the ROI, the calibration image can be converted to a spectral form as a function of energy as shown in Fig. 1 ▸(*b*). Here, the *y*-axis represents the total photons counted within eight ROIs. The 20 peaks in Fig. 1 ▸(*b*) correspond to the collected monochromatic X-ray photons, and the HWHM of each peak. The average of the 20 HWHMs is defined as the instrumental broadening originating from experimental errors in this study, and the average HWHMs and standard deviation of all samples were estimated as listed in Table 1 ▸, which were used in the convolution process.

## 3. *AXEAP2* features

**Figure 2:**
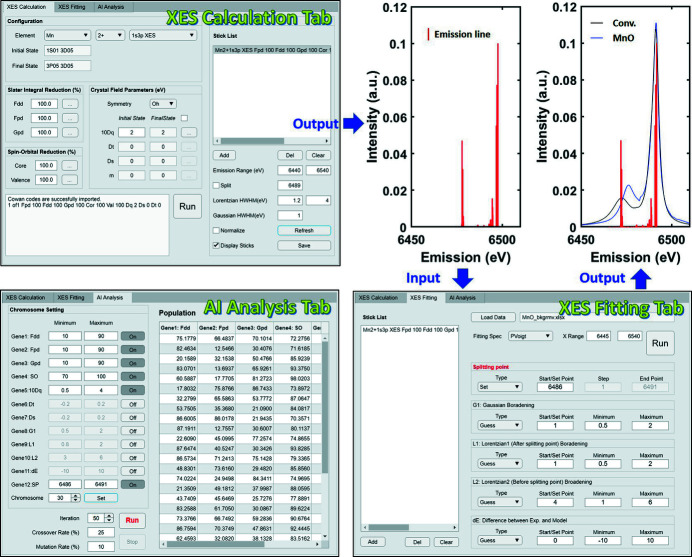
Screen shots of the three modules in *AXEAP2* (XES calculation, XES fitting and AI analysis tabs) and two example output plots. The red vertical lines on the two input plots represent emission probabilities. The blue line and black line are the MnO *K*β XES spectrum and the spectrum convoluted by the XES fitting tab using vertical lines, respectively.

Our methodology and *AXEAP2* can be divided into three parts: XES calculation, XES fitting and parameter optimization. These three parts are provided as modules within the main window of *AXEAP2*, accessible via a series of interface windows in the control panel, as shown in Fig. 2 ▸.

The XES calculation module provides the ability to perform theoretical XES calculations with input parameters. This module was created by extracting and adapting the source codes of *CTM4XAS* related to XES calculation, ensuring familiarity and ease-of-use for those experienced with *CTM4XAS*, as the interface closely resembles that of *CTM4XAS*.

The XES fitting module is designed to fine-tune the theoretically calculated emission energy and its probability to match the shape of experimental spectra through the use of non-linear least square (NLS) fitting. This module supports Gauss, Lorentz and pseudo-Voigt functions.

Finally, the AI analysis module provides the ability to optimize the parameters used in XES calculation and XES fitting by specifying the optimization range and defining the required values. Upon completion of the optimization process, the results of the calculation and data analysis can be reviewed and confirmed through the GA viewer.

### 3.1. XES calculation

The theoretical approach in *CTM4XAS* is described thoroughly elsewhere (Cowan, 1981 ▸; de Groot & Kotani, 2008 ▸; Stavitski & de Groot, 2010 ▸). In this paper, a brief overview of the parameters is given.

The starting point of the XES calculation is to consider the electron–electron interaction and spin–orbital coupling for all electrons in an atom. The electron–electron interaction is described using Slater integrals. The Slater integrals are calculated within the Hartree–Fock limit and controlled by the first reduction factor. In this study, the reduction factors are expressed as *F*_dd_ (3*d*–3*d* Coulomb interaction), *F*_pd_ (3*d*–3*p* Coulomb interaction) and *G*_pd_ (3*d*–3*p* exchange interaction).

The next step is to consider the spin–orbital term including core level and valence level. *CTM4XAS* defines the spin–orbital energy reduction factor as *SO* in an equivalent manner as the Slater integral reduction factors. We only consider the part of the valence *SO* optimization parameter because it is assumed that samples studied here have no screening effect on the core level.

The final step is to add a crystal field potential effect. This requires three input variables, which *CTM4XAS* describes as *10Dq*, *Dt* and *Ds* (Solomon & Lever, 1999 ▸). Varying *10Dq*, *Dt* and *Ds* in various symmetries such as octahedral (*O*_*h*_), tetragonal (*D*_4*h*_) and fourfold (*C*_4_) can yield three spin states: a high spin (HS), an intermediate spin (IS) and a low spin (LS). In the *CTM4XAS* calculation, spin state is determined by *10Dq* and the pairing energy (or exchange splitting). The pairing is present for every two parallel electrons and can be approximated by a linear combination of the Slater integrals related with *F*_dd_ (de Groot, 2005 ▸). The spin state is a matter of great attention in *K*β XES because HS tends to have a prominent *K*β′ feature relative to LS or IS. The crystal field parameters, therefore, were treated as optimization targets in the same way as atomic parameters.

### 3.2. XES fitting

In general, when the atomic multiplet theory, spin–orbital effect and crystal field effect are considered in the *K*β mainline calculation for 3*d* transition metals, the *CTM4XAS* program generates up to 100 or more discrete emission probabilities. Convolution can be understood as applying core-hole lifetime and instrumental broadening by applying Lorentzian (*L*) and Gaussian (*G*) functions to emission probabilities. To reflect both *L* and *G* in the spectrum, the pseudo-Voigt function (*V*_p_) is widely used (Ida *et al.*, 2000 ▸). *V*_p_ is given by

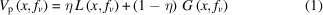

with 0 < η < 1, where η is a function of the full width at half-maximum (FWHM) parameter given by

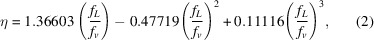

where *f*_*L*_ and *f*_*G*_ are the FWHM of *L* and *G*, respectively (Ida *et al.*, 2000 ▸). *f*_*v*_ is defined as the FWHM of *V*_p_ and is described by

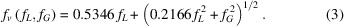

As previously noted, *K*β_1,3_ and *K*β′ splitting arises from core-to-core or core-to-valence electron interactions in a transition metal element, and they have different final states. In other words, different Lorentzians should be applied to each emission probability. However, since it is difficult to apply different broadening to 100 or more probabilities as well as theoretical core-hole life calculations, an alternative method of applying different widths from a point called the splitting point (SP) is used (Kucheryavy *et al.*, 2016 ▸; Peng *et al.*, 1994 ▸). For example, a broadening value *L*_1_ is applied to the emission probability above SP, and *L*_2_ is applied to the emission probability below SP. This is described by

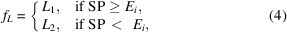

where *E*_*i*_ is an *i*th emission energy.

The analysis of *K*β mainlines does not reflect the absolute intensity – only relative intensities and the energy range between *K*β_1,3_ and *K*β′ are needed (Vankó *et al.*, 2006 ▸). To allow all generated emission probabilities to be adjusted equally according to the normalized spectral intensity, a parameter *I*_0_ is defined. In this study the optimized results of *I*_0_ are not presented. The *dE* parameter was adopted to correct the difference that occurs in the calibration process or in the calculation.

In summary, to apply the convolution, six parameters must be defined: Gaussian HWHM (*G*_1_), splitting point (*SP*), Lorentzian HWHM after *SP* (*L*_1_), Lorentzian HWHM before *SP* (*L*_2_), energy difference between experiment spectrum and theoretical spectrum (*dE*), and overall emission intensity adjustment factor (*I*_0_). To ensure that the emission spectrum calculated in theory is generated in close proximity to the experimental spectrum, the NLS fitting method was introduced.

### 3.3. AI analysis

**Figure 3:**
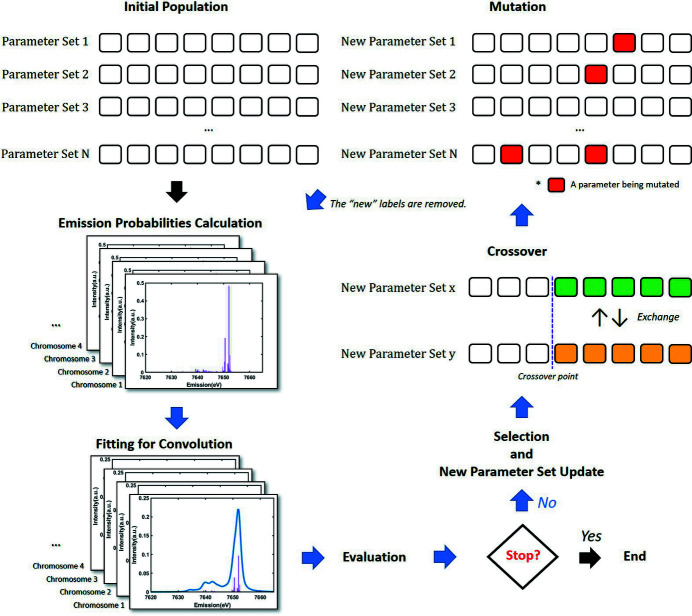
Workflow of the GA-based XES analysis.

Fig. 3 ▸ shows the workflow for XES parameter optimization. A parameter to be optimized in the GA is defined as a gene. The atomic parameters and the crystal field parameters are genes, while the convolution parameters can optionally be either genes or fitting parameters. A set of genes is referred to as a chromosome and a set of chromosomes is defined as a population. 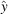 can be defined as a spectrum function *S* resulting from the emission probabilities after the convolution through the NLS fitting,

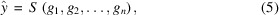

where *g*_1_, *g*_2_,…, *g*_*n*_ are genes in a chromosome. The identification strategy is to find the best chromosome to minimize the difference between experimental XES spectra and 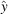 . The root-mean-square error (RMSE) is the most appropriate indicator for this and is represented by

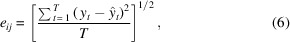

where *e*_*ij*_ is the RMSE of the *j*th chromosome on the *i*th population, and *y*_*t*_ is an experimental spectrum. Selection probability (*F*_*ij*_) helps to create a next generation based on randomly selected chromosomes from the previous generation, which is called fitness (Mitchell, 1996 ▸). Then the fitness is defined and estimated by

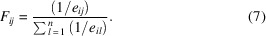

The defined fitness increases the probability that a chromosome is selected when the fitness is high.

Selecting chromosomes adopted the roulette wheel method. The area of each chromosome on the roulette board directly reflects the fitness score. The number of chromosomes in all generations is the same because roulette always operates as many chromosomes in a population. Chromosomes can be chosen in duplicate, so if there is a chromosome with an overwhelmingly high fitness score it becomes dominant.

The next step is the crossover. In the previous step, some chromosomes are randomly selected within a population. The selected chromosomes are new chromosomes for the next generation, but they may also have a new combination of genes by exchanging genes (Mitchell, 1996 ▸). The exchange of genes between new chromosomes is called crossover. The use of crossover, therefore, can enable GA to search the entire parameter space in parallel, helping it avoid getting trapped in local maxima. The genes to be exchanged are randomly selected within a range that does not exceed the total number of genes in a chromosome.

There is another way to avoid getting trapped in local maxima, namely mutation. The crossover step produces a variety of gene arrangements. Since the selection roulette allows for duplicate selection, the gene pool would tend to become more and more homogeneous as one gene begins to dominate over generations (Mitchell, 1996 ▸). Mutation remedies the homogeneity by introducing genetic diversty, *i.e.* replacing genes with new values. This swap of genes also enables the GA to explore a large portion of the parameter space beyond that spanned by the initial population. With each iteration (or population) of the algorithm, some genes in a chromosome will be mutated within a gene’s limitation.

### 3.4. GA viewer

During optimization, *AXEAP2* files all relevant information such as emission probabilities, fitting results and parameters for each spectrum. The GA viewer facilitates the visualization of this information, providing a comprehensive understanding of the results.

The uncertainties of parameters used in NLS fitting can be directly estimated, but the uncertainties of genes cannot be directly obtained. This is because the fitness of a chromosome can only be confirmed after convolution with the NLS fitting process. Instead, the GA viewer supports scatter plots for the parameters generated during GA iteration. GA is based on selection rules such as biological evolution, and, the higher the fitness, the more likely the chromosome is to be inherited by the next generation. The scatter plot is designed to help scientists see the distribution of genes and make inferences about them. An example of using this function is presented in the *Results* [Sec sec5] section.

### 3.5. Programming environment

*AXEAP2* is written in MATLAB2021b and works only on Windows OS. This program is freely available, by email. In order to run the program, MATLAB Runtime 2021b is required – a standalone set of shared libraries that enables the execution of compiled *MATLAB* applications or component without license. MATLAB Runtime can be accessed via the MathWorks website (https://www.mathworks.com/products/compiler/mcr/index.html).

## 4. Application of the GA to experimental data

Prior to optimization, all experimental spectra were normalized to have an integral area of 1 within the effective energy range. The orbital configuration of samples during 3*p* → 1*s* transition was determined by the number of electrons as listed in Table 1 ▸. For example, Mn^2+^ (1*s*3*d* ^5^ → 3*p* ^5^3*d* ^5^) and Mn^3+^ (1*s*3*d* ^4^ → 3*p* ^5^3*d* ^4^) configurations are used for MnO and Mn_2_O_3_, respectively. For LiNiO_2_, since the electronic structure of the Ni ions is still under debate and it is unclear whether it is HS divalent or LS trivalent, two configurations were applied for comparison (Sicolo *et al.*, 2020 ▸; Montoro *et al.*, 1999 ▸). The parameters *F*_dd_, *F*_pd_, *G*_pd_, *10Dq*, *SO* and *SP* were set as genes, and *O*_*h*_ symmetry was used.

To convolute the XES calculation results, we chose the pseudo-Voigt function, which can reflect instrumental and theoretical broadening as described in Section 3.2. The value of the experimental broadening parameter *G*_1_ for each sample was set to the HWHM listed in Table 1 ▸, and the upper and lower limits of each parameter are shown in Table 2 ▸.

The crossover and mutation rate should be adjusted so that appropriate crossover and mutations can occur in one generation. Excessive crossover and mutations make optimization difficult due to frequent variable changes before becoming dominant, while, in the opposite case, genes are trapped in local optimization and cannot escape easily. Appropriate values for population size, crossover and mutation rates were found and applied through several trials. The use of 20% to 30% for crossover and 10% to 15% for mutation showed stable optimization, and the default values for crossover and mutation rates in *AXEAP2* were set to 25% and 10%, respectively. In this study, we utilized the default values set in *AXEAP2* (Hermawanto, 2013 ▸). In addition, the number of chromosomes in a population and iterations were set to 30 and 50, respectively, to generate enough data for optimization. It took about 1 h for a desktop computer with a single core of Intel 10th Generation i7 CPU to optimize a spectrum by the above description.

## 5. Results

**Figure 4:**
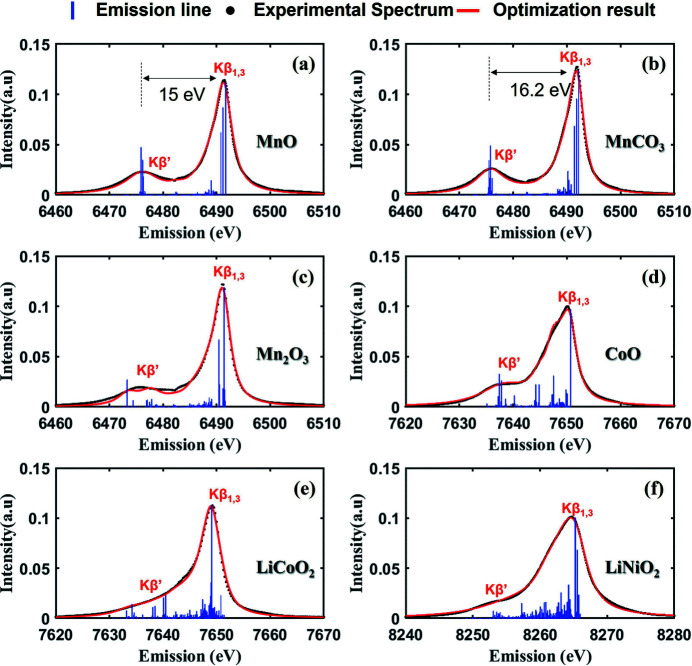
The best results of GA-based optimization for (*a*) MnO, (*b*) MnCO_3_, (*c*) Mn_2_O_3_, (*d*) CoO, (*e*) LiCoO_2_ and (*f*) LiNiO_2_. The dotted black line, the solid red line the and blue bar represent the experiment data, optimized spectrum and emission probability, respectively.

In this study, the criterion for an acceptable optimization result was defined as achieving lower than the mean RMSE among populations. The MnO, MnCO_3_, Mn_2_O_3_, CoO, LiCoO_2_ and LiNiO_2_ spectra and lowest RMSE model are presented in Fig. 4 ▸. The parameters for the model with lowest RMSE are listed in Table 3 ▸.

**Figure 5:**
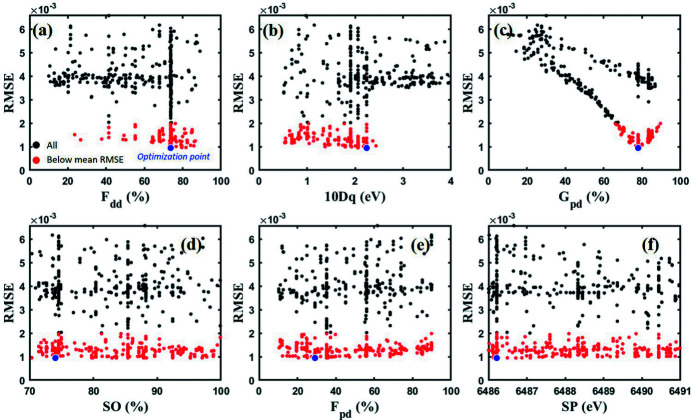
Scatter plot of (*a*) *F*_dd_, (*b*) *10Dq*, (*c*) *G*_pd_, (*d*) *SO*, (*e*) *F*_pd_ and (*f*) *SP* genes of MnO. The red dots represent genes below the mean RMSE and the blue dot represents the optimized value found by *AXEAP2*.

It is important to note that our method does not provide uncertainty for the gene type parameter. Instead, an alternative method was used to analyze the relationship between the RMSE and all parameters generated during the optimization process by comparing them. An example of this is shown in Fig. 5 ▸, which displays scatter plots of MnO genes. Points represent all genes generated across all populations, with red markers indicating values with lower than the mean RMSE among all optimized fitting models, which are considered acceptable results of the fitting optimization process.

**Figure 6:**
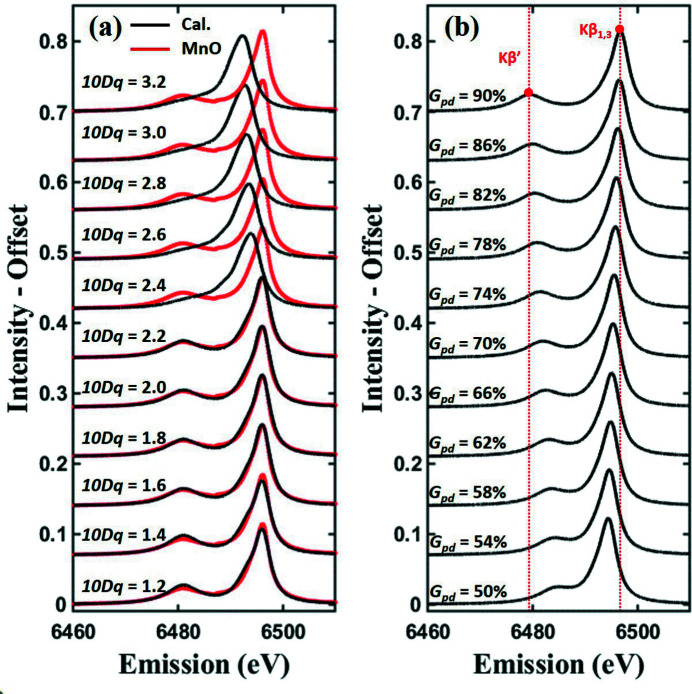
Calculated spectra of Mn^2+^ showing the effect of (*a*) *10Dq* and (*b*) *G*_pd_ values. Other parameters are set to optimized values of MnO as listed in Table 3 ▸. The red lines in (*a*) are the same MnO spectrum shown in Fig. 4 ▸(*a*) and the black lines in (*a*) and (*b*) are calculated spectra. The red dotted vertical lines in (*b*) are a guide to the eye.

As shown in Figs. 5 ▸(*a*) and 5(*b*), the red *F*_dd_ points are primarily located above the 60% *F*_dd_ value, while the red *10Dq* points are concentrated below 2.20 eV. The pairing energy of divalent Mn without reduction is about 3.14 eV, as calculated using *AXEAP2*. When the lowest RMSE *F*_dd_ value of MnO is applied, the pairing energy is about 2.31 eV (3.14 × 0.737), which is larger than *10Dq*, so all five electrons of MnO are aligned in spin-up, resulting in HS. In the *O*_*h*_ symmetry, the theoretical XES spectrum becomes HS when the condition (pairing energy) × (*F*_dd_/100) > *10Dq* is satisfied. Fig. 6 ▸(*a*) shows the effect of the spin state by applying the optimized value of MnO listed in Table 3 ▸ and changing the *10Dq* values. If the *10Dq* value is less than 2.31 eV, which is the electron pairing energy, all become HS. The HS spectra exhibit relatively better fits with RMSE values less than the mean when compared with LS, thus creating empty spaces in the *F*_dd_ and *10Dq* scatter plots corresponding to LS.

The overall distribution of *G*_pd_ points in Fig. 5 ▸(*c*) is V-shaped and converged to 77.9% of the *G*_pd_ value, meaning that *G*_pd_ has a strong effect on spectral shape and is well defined by *AXEAP2*. In fact, Fig. 6 ▸(*b*) shows the effect of the *G*_pd_ value. As the *G*_pd_ value increases, the *K*β_1,3_ and *K*β′ peaks become farther apart. Other parameters *SO*, *F*_pd_ and *SP* show no significant relationship to RMSE as shown in Figs. 5 ▸(*d*), 5(*e*) and 5(*f*), respectively, and it was difficult to find a clear role in the optimization results of all samples. Therefore, these values are not determined by *AXEAP2*.

The MnCO_3_ spectrum shows a clear separation of *K*β_1,3_ and *K*β′, similar to the separation in MnO, and the energy difference between the *K*β_1,3_ and *K*β′ of MnCO_3_ is about 1.2 eV larger than that of MnO. In addition, when the intensities of the *K*β_1,3_ and *K*β′ peaks are compared by normalizing the two spectra, the peak intensity of MnCO_3_ is higher than that of MnO. Since MnCO_3_ has the same number of electrons as MnO, the pairing energy is the same as that of MnO when there is no reduction. The lowest RMSE reduction factor of the *F*_dd_ value is 81.5%, and, when this is applied, the final pairing energy is about 2.56 eV (3.14 × 0.815), which is higher than the *10Dq* value of 2.54 eV for MnCO_3_. This means that MnCO_3_ has HS with the same spin alignment as MnO.

The experimental results of Mn_2_O_3_ show that the *K*β′ intensity is weaker than that of MnO, and a mismatch is found between *K*β′ experimental and optimization results. Also, *L*_2_ is significantly lower than other optimization results. The pairing energy of trivalent Mn was calculated using the same method as for MnO and was found to be approximately 2.73 eV (3.25 × 0.841) in Mn_2_O_3_. This value is higher than the *10Dq* value of 2.24 eV for Mn_2_O_3_, resulting in HS.

The experimental CoO spectrum has a higher-intensity *K*β′ peak than LiCoO_2_. The Co ions in CoO and LiCoO_2_ are known to have seven and six electrons in the 3*d* orbital, respectively. Interestingly, CoO and LiCoO_2_ had low *10Dq* values, but *AXEAP2* predicted CoO as HS and LiCoO_2_ as LS. So, four electrons in CoO are paired and three electrons are aligned in spin-up, whereas the six electrons in LiCoO_2_ are all paired.

The *K*β peak width of LiNiO_2_ is wider than other measured spectra and *L*_1_ and *L*_2_ also are the highest. As described previously, for this material it is not clear whether the Ni ion is divalent or trivalent. When the divalent and trivalent Ni configurations were applied to LiNiO_2_, the LS trivalent model was found to be closer. This means that six electrons are paired and one electron is aligned in spin-up. As with LiCoO_2_, an *F*_dd_ of 32.5% is small when multiplied by the pairing energy to give a value lower than the low *10Dq* value for LS.

The uncertainty of *L*_1_, *L*_2_ and *dE* can be directly estimated by NLS fitting, indicating a high level of precision and confidence in the results with small uncertainties. The parameter *dE*, which represents the difference between the optimized spectrum and the experimental spectrum, shows a similar difference when the element species is the same. Negative-optimized values as listed in Table 3 ▸ indicate that the energy of the experimental spectrum is shifted to higher energy, compared with the spectrum calculated by *AXEAP2*.

## 6. Discussion

*AXEAP2* was tested using six spectra to evaluate its performance and capabilities. This program does not provide uncertainty on the parameters used as a gene, so an alternative method was used to understand the results using a comparison method based on the relationship between RMSE and parameter. Theoretical spectra were generated as close as possible to the experimental spectra within the physical/chemical boundaries. A range of values were found with acceptable RMSE values as shown in the scatter plots of Fig. 5 ▸ for *F*_dd_, *10Dq* and *G*_pd_. The values for *SO*, *F*_pd_ and *SP* did not have a significant effect on the data and were not determined.

The theoretical spin state was determined by comparing the spin pairing energy with the optimized *F*_dd_ and *10Dq* values based on the atomic multiplet theory and crystal field effect. Some of our samples have lowest RMSE *10Dq* values that are similar to values calculated or used in previous literature, but there are also *10Dq* values that differ significantly. The *AXEAP2* algorithm reports the lowest RMSE value, although the scatter plots show a range with similar RMSE values that need to be considered. For example, *10Dq* values for MnO and MnCO_3_ have been previously reported to be in the range 1.0–1.4 eV (Qiao *et al.*, 2013 ▸; de Groot *et al.*, 1989 ▸; Glatzel *et al.*, 2004 ▸). *AXEAP2* reports the lowest RMSE value of 2.2 to 2.5 eV (blue dot in Fig. 5 ▸) and a range of acceptable values from 0.5 to 2.5 (red dots in Fig. 5 ▸). The scatter plots show the lowest RMSE value at one end of this range but with little differentiation between the RMSE values throughout the range. As shown in Fig. 5 ▸, points satisfying (pairing energy) × (*F*_dd_ / 100) > *10Dq* for HS include both the literature reported values and the lowest RMSE values. Figs. 5 ▸(*a*) and 5(*b*) can be interpreted as a strong trend of making HS for the MnO spectrum by lowering the *10Dq* value and increasing the *F*_dd_ value. The spin states of the six samples were predicted and the predicted results were consistent with the references, except for LiNiO_2_ where the valency is not yet clear. A clue that the *G*_pd_ value played a decisive role in a spectral shape was found in a comparison of the scatter plot and the spectral shapes with different *G*_pd_ values, as shown in Figs. 5 ▸ and 6 ▸(*b*). The low uncertainty of *L*_1_ and *L*_2_ indicates that the effect on the core-hole lifetime can be estimated numerically, if the misfit of the spectrum is not large.

### 6.1. MnO and MnCO_3_

Previous studies have shown that both materials have the same number of electrons, valency and spin state. The results of spin states predicted by *AXEAP2* are also consistent with previous studies. What is noteworthy is that the energy difference between *K*β_1,3_ and *K*β′ in the two spectra appears differently despite having the same electron configuration. Pollock *et al.* (2014 ▸) investigated the characteristics of the *K*β_1,3_ and *K*β′ emission spectra. They utilized the restricted active space configuration interaction and crystal field multiplet calculations to determine the decisive parameters in shaping the appropriate broadening of the spectra. They found that the 3*p*–3*d* exchange interaction parameters have major impact on the spectral shape when the spin state is preserved, while other parameters have minimal influence. Their work provides a reasonable explanation for why the *G*_pd_ value is the most sensitive compared with other parameters in our findings, suggesting that the 3*p*–3*d* exchange force plays a key role in contributing to the energy difference between *K*β_1,3_ and *K*β′ even in MnO and MnCO_3_ spectra with the same electron number and configuration. In fact, the *G*_pd_ value of MnCO_3_ is higher than that of MnO as listed in Table 3 ▸.

### 6.2. MnO (or MnCO_3_) and Mn_2_O_3_

The number of electrons in trivalent Mn is 4, which is one less than for divalent Mn such as MnO. As the number of unpaired electrons increases, the intensity of the *K*β′ line is known to be relatively low even though it has the same spin state. This fact is well reflected on experimental spectra of MnO and Mn_2_O_3_ and the optimization spectra also show that the overall emission probabilities of trivalent Mn in the *K*β′ region is lower than that of the divalent Mn model. However, a discrepancy around the *K*β′ region was found between experiment and GA optimization. The discrepancy can be understood in two ways: either the optimization process has not reached the best values during the limited iteration, or the calculation model has not reached optimal state. The first hypothesis was ruled out through ten additional optimizations, all of which produced similar results. Thus, it is likely that the model was not appropriate for the experimental data. For this reason, the low *L*_2_ value of Mn_2_O_3_, which represents the core-hole broadening effect of the *K*β′ line, can be interpreted as an optimized result that attempts to fit the spectral line as closely as possible.

### 6.3. CoO and LiCoO_2_

The comparison of the two spectra represents conditions in which a different spin state is introduced in addition to comparison conditions of MnO and Mn_2_O_3_. The predicted spin states are consistent with the references, leading to more dramatic changes in *K*β′ due to the spin state difference. The *10Dq* value of 0.7–1.1 eV for CoO can be found in the previous works, but the *10Dq* value of 0.86 eV for LiCoO_2_ is very low compared with the value (∼2.4 eV) obtained by experiments and calculation (van Elp *et al.*, 1991 ▸; Ensling *et al.*, 2014 ▸; Iida *et al.*, 2021 ▸). Furthermore, *F*_dd_ for LiCoO_2_ was found to be a very low value, which is a result of constructing a lower pairing energy than the *10Dq* value. This means that *F*_dd_ and *10Dq* are more focused on the spin state during optimization and may differ significantly from the actual value, similar to the MnO and MnCO_3_ cases, as described above.

### 6.4. LiNiO_2_ and CoO

*AXEAP2* concludes that LiNiO_2_ is close to trivalent LS. The *L*_1_ and *L*_2_ values of LiNiO_2_ are larger than for other spectra, which reflects the relatively wide widths of the *K*β mainlines. Several studies have investigated core-hole broadening in the *K*α and *K*β lines of the 3*d* transition metals and reported that the width of the spectrum widens as the atomic number increases (Krause & Oliver, 1979 ▸; Hölzer *et al.*, 1997 ▸; Ito *et al.*, 2018 ▸). These differences affect the shape of the two spectra, and the area around *K*β′ in particular shows the difference between HS and LS, even though both spectra have the same number of electrons.

## 7. Summary and conclusion

In this study, we have proposed a method for optimizing the XES parameters using a GA-based algorithm and matching the spectrum between the experimental data and the theoretical model. We set six genes, 30 chromosomes in a population, 25% crossover rate, 10% mutation and 50 iterations per experiment data set. The XES calculation part in the *CTM4XAS* program was extracted and re-coded into a friendly package that is easy to use. To convolute the result of the *CTM4XAS* calculation, a pseudo-Voigt function and NLS fitting were introduced. Although a discrepancy was found in the *K*β′ of Mn_2_O_3_, the overall spectral appearance of the optimized result is very similar to the experimental data within the range of physical and chemical limitations. Also, *AXEAP2* has merit in that it can numerically estimate the spin state, 3*d*–3*p* electron exchange force, and the broadening effect that arises from the core-hole lifetime, which is difficult to obtain information. Therefore, *AXEAP2* will be of great help in finding an acceptable range of parameters that most strongly determine the spin state and *G*_pd_ when the experimental environment changes, *i.e.* experimental results with pressure or temperature changes, have not been reported before. Although a GA-based algorithm requires high computational costs, it will be very helpful from the perspective of reducing trial-and-error approaches.

## Figures and Tables

**Table 1:** Sample information and their experimental energy resolution Std is the standard deviation of the HWHM.

Sample	Analyzer	HWHM (eV)	Std (eV)	Formal valence	3*d* occupancy	Spin state	Symmetry
MnO	Ge (044)	0.393	0.009	2+	3*d* ^5^	HS	*O*_*h*_
MnCO_3_	Ge (044)	0.425	0.016	2+	3*d* ^5^	HS	*O*_*h*_
Mn_2_O_3_	Ge (044)	0.414	0.007	3+	3*d* ^4^	HS	*O*_*h*_
CoO	Ge (444)	0.470	0.007	2+	3*d* ^7^	HS	*O*_*h*_
LiCoO_2_	Ge (444)	0.492	0.034	3+	3*d* ^6^	LS	*O*_*h*_
LiNiO_2_	Si (444)	0.552	0.013	2+ or 3+	3*d* ^8^ or 3*d* ^7^	HS or LS	*O*_*h*_

**Table 2:** List of parameters used in GA and NLS fitting

Parameter	Lower bound	Upper bound	Description
*F*_dd_	10%	90%	Reduction factor of 3*d*–3*d* electrons Coulomb force
*F*_pd_	10%	90%	Reduction factor of 3*p*–3*d* electrons Coulomb force
*G*_pd_	10%	90%	Reduction factor of 3*p*–3*d* electrons exchange force
*SO*	70%	100%	Spin–orbital coupling reduction factor
*10Dq*	0.5 eV	4.0 eV	Crystal field energy
*Dt*	–		Crystal field energy
*Ds*	–		Crystal field energy
*SP*	*K*β mainline − 5	*K*β mainline + 1	Splitting point to apply different broadening
*G*_1_	Instrumental broadening (Section 2)		Gaussian half width at half-maximum (HWHM)
*L*_1_	1.0 eV	3.0 eV	Lorentzian HWHM after SP
*L*_2_	2.0 eV	8.0 eV	Lorentzian HWHM before SP
*dE*	−10 eV	+10 eV	Energy difference between experiment and theory
*I*_0_	0.0	1.0	Overall emission intensity adjustment factor

**Table 3:** Pairing energies calculated by *AXEAP2* and parameter values from the lowest RMSE gene

	Pairing energy (eV)	*F*_dd_ (%)	*10Dq* (eV)	Spin state	*G*_pd_ (%)	*F*_pd_ (%)†	*SO* (%)†	*SP* (eV)†	*L*_1_ (eV)	*L*_2_ (eV)	*dE* (eV)
MnO	3.14	73.7	2.24	HS	77.9	29.1	74.0	6486.2	1.64 ± 0.01	3.70 ± 0.06	−4.69 ± 0.01
MnCO_3_	3.14	81.5	2.54	HS	84.4	83.3	89.6	6489.9	1.32 ± 0.01	3.05 ± 0.07	−4.49 ± 0.01
Mn_2_O_3_	3.25	84.1	2.24	HS	76.6	22.4	84.6	6489.0	1.46 ± 0.03	2.02 ± 0.19	−4.29 ± 0.01
CoO	2.80	32.3	0.82	HS	70.6	86.1	94.6	7648.0	1.37 ± 0.02	4.61 ± 0.11	−3.67 ± 0.01
LiCoO_2_	2.46	28.0	0.86	LS	81.0	62.0	80.4	7644.4	1.51 ± 0.01	3.29 ± 0.25	−3.40 ± 0.01
LiNiO_2_	2.92	56.2	2.40	LS	58.2	52.5	98.5	8264.4	1.84 ± 0.01	4.89 ± 0.16	−4.04 ± 0.01

† No dependence on the optimization process.
